# Isolated Cobalt Ions Embedded in Magnesium Oxide Nanostructures: Spectroscopic Properties and Redox Activity

**DOI:** 10.1002/chem.202002817

**Published:** 2020-10-19

**Authors:** Thomas Schwab, Matthias Niedermaier, Gregor A. Zickler, Milan Ončák, Oliver Diwald

**Affiliations:** ^1^ Department of Chemistry and Physics of Materials Paris-Lodron University Salzburg Jakob-Haringer-Straße 2a 5020 Salzburg Austria; ^2^ Institute for Ion Physics and Applied Physics University Innsbruck Technikerstraße 25 A-6020 Innsbruck Austria

**Keywords:** doping, interfacial charge transfer, isolated transition metal ions, oxide nanocrystals, single-site catalysis

## Abstract

Atomic dispersion of dopants and control over their defect chemistry are central goals in the development of oxide nanoparticles for functional materials with dedicated electronic, optical or magnetic properties. We produced highly dispersed oxide nanocubes with atomic distribution of cobalt ions in substitutional sites of the MgO host lattice via metal organic chemical vapor synthesis. Vacuum annealing of the nanoparticle powders up to 1173 K has no effect on the shape of the individual particles and only leads to moderate particle coarsening. Such materials processing, however, gives rise to the electronic reduction of particle surfaces, which—upon O_2_ admission—stabilize anionic oxygen radicals that are accessible to UV/Vis diffuse reflectance and electron paramagnetic resonance (EPR) spectroscopy. Multi‐reference quantum chemical calculations show that the optical bands observed mainly originate from transitions into ^4^A_2*g*_ (^4^F), ^4^T_1*g*_ (^4^P) states with a contribution of transitions into ^2^T_1*g*_, ^2^T_2*g*_ (^2^G) states through spin‐orbit coupling and gain intensity through vibrational motion of the MgO lattice or the asymmetric ion field. Related nanostructures are a promising material system for single atomic site catalysis. At the same time, it represents an extremely valuable model system for the study of interfacial electron transfer processes that are key to nanoparticle chemistry and photochemistry at room temperature, and in heterogeneous catalysis.

## Graphical abstract

**A MgO host lattice** with an atomic distribution of cobalt ions in substitutional sites was achieved via metal organic chemical vapor synthesis, providing a promising material system for single atomic site catalysis and a good model system for the study of interfacial electron transfer processes that are key to nanoparticle chemistry and photochemistry at room temperature, and in heterogeneous catalysis.

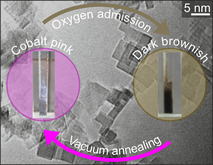

## Introduction

Metal oxide nanoparticles having well‐defined surface structures and compositions are versatile building blocks for their controlled functionalization with organic^[1]^ and/or inorganic surface groups.^[2]^ Uniformly distributed particle characteristics, such as a narrow particle size distribution in conjunction with a characteristic crystal habit that is made up by only few types of low energy planes render such nanoparticle systems well‐suited for further integration into hierarchical structures. Their organization into hybrids can lead to a high level of order at larger length scales.^[3]^

The admixture of impurities to introduce additional properties to the functional particle system, such as redox‐activity,^[4]^ color or magnetism,^[5]^ complicates the structural situation due to changes in the defect chemistry of the particle. Moreover, segregation effects or dopant induced surface energy changes can occur.^[6]^ The chemical nature of the impurity ions, their ionic radius as substitutional dopants and their redox activity are indispensable for the prediction of associated property changes the nanoparticles undergo in the course of annealing and surface chemical reactions. Prototypical model systems for the study of doping effects on insulating metal oxides are related to transition metal ions in micro‐ or single‐crystalline MgO.^[7]^ Substantial interest for these systems has originated from the idea to transfer the well‐understood mass transport mechanisms of alkali halides to magnesium oxide due to its simple cubic rock salt structure.^[8]^ Hence, it is important to rationalize the dispersion and properties of impurities and to predict their influence on structure and stability of surfaces and interfaces.^[9]^ To engineer ionic and electronic conductivity contributions[^7b^, ^10^] or to adjust creep behavior or sinterability have been important motivations to dedicate related studies to undoped and doped MgO structures.^[7a]^ This also applies for the advancement of our understanding of complex catalysts during heterogeneous catalysis.^[11]^ Examples for fundamental mechanistic studies in heterogeneous catalysis are the investigation of Fe‐doped catalysts for the oxidative coupling of methane reaction,^[12]^ the investigation of surface chemistry of Ni‐doped MgO^[13]^ or the investigation of Co‐doped MgO powders for the conversion of nitric oxides and or for alkene oxidation.^[14]^

Many of these studies made use of molecular spectroscopy techniques like UV/Vis diffuse reflectance, photoluminescence (PL) or electron paramagnetic resonance (EPR) spectroscopy and achieved at the atomic/molecular level detailed insights into electronic structure changes that arise from doping and impurity admixture. Spectroscopy studies on metal oxide particle systems, however, typically suffer from the broad distribution of geometrically and electronically different sites leading to less‐defined spectroscopic features of lower intensities. Systems of particles that adopt a simple shape and exhibit uniform particle properties are therefore indispensable for a correct assignment of distinct signals to specific surface elements, such as corners, kinks and edges, and associated adsorbates.

This study makes use of a system of doped MgO nanocubes where recent XAS and XPS studies in combination with results from ab initio calculations[^6^, ^15^] provided detailed information about the uniform distribution of Co^2+^ ions over the cationic sublattice. Here, we report the spectroscopic changes Co‐doped MgO nanocube powders can undergo during interfacial redox reactions at room temperature. The possibility to re‐achieve electronic reduction of the material by vacuum annealing in combination with their high thermal stability makes respective particle powders to extremely well‐suited model substrates for the elucidation of mechanistic details in heterogeneous oxidation catalysis or for sensing applications where a tuned redox activity can be linked to the adsorption of complex organic molecules.

## Experimental Methods and Computations

**Particle synthesis**: Co‐Mg‐O nanoparticles were synthesized using a hybrid hot wall reactor system, as described in refs. [11b, 16]. A cobaltocene (Sigma Aldrich, 0.5 g) precursor positioned inside the inner quartz glass tube was heated up to temperatures in the range between *T*_1_=318 K and 333 K in order to sublimate the metalorganic precursor with a well‐adjusted evaporation rate. An argon flow (Ar 5.0, volumetric flow rate *Q*_Ar_=1200 sccm) transports the cobaltocene vapor to a position where heating of Mg metal turnings (99.98 %, Alfa Aesar, 1.0 g) leads to Mg sublimation and mixing with the gaseous cobaltocene precursor. Further transport of the vapor mixture leads to contact with molecular oxygen (O_2_ 5.0, *Q*_O2_=1200 sccm). Subsequent cobaltocene precursor decomposition inside the Mg combustion flame leads to homogeneous nucleation and nanoparticle formation in the gas phase. The MgO based nanoparticle powder agglomerates are collected down stream the reactor inside a stainless steel net at room temperature. Temperatures of operation and the total pressure (*p=*70±2 mbar) are kept constant during the entire process of particle collection. Further details are provided in references [15, 16].

**Transmission electron microscopy (TEM)**: TEM data were acquired using a JEOL JEM‐F200 transmission electron microscope equipped with a cold field emission electron source and a large windowless JEOL Centurio EDX (Energy Dispersive X‐ray emission) detector (100 mm^2^, 0.97 srad, energy resolution <133 eV) for local composition analysis, operating at 200 kV. Admixture concentrations were obtained by applying the k‐factor method on the EDX counts of the Mg K_α_ transition line for Mg (1.253 keV) and the Co K_α_ line for Co (6.924 keV). TEM images were recorded using a TVIPS F216 2k by 2k CMOS camera. TEM grids were prepared by dipping a lacey carbon grid into the powder in order to investigate structural features and composition of material sticking to the carbon grid.

**Ultraviolet‐visible (UV/Vis) spectroscopy**: UV/Vis spectra in diffuse reflectance were acquired with a PerkinElmer—Lambda 750 UV/Vis/NIR spectrophotometer, equipped with an integrating sphere (*d=*60 mm, Spectralon) and converted to absorption spectra by Kubelka‐Munk transformation. An optical high vacuum tight fused silica cell was used for spectroscopic investigation and allows for optical measurements at pressures as low as *p*(O_2_) <10^−5^ mbar or in defined gas atmospheres. Spectra were acquired under static high vacuum conditions prior to oxygen admission (*p*(O_2_)=100 mbar) and after subsequent evacuation down to *p*(O_2_) <10^−5^ mbar to track O_2_ adsorption induced changes on the optical absorption properties.

**Electron paramagnetic resonance (EPR) spectroscopy**: For EPR measurements, the powder samples were transferred into a Suprasil quartz glass tube (*d=*5 mm) and connected to an appropriate high vacuum pumping system with a base pressure as low as *p*(O_2_) <10^−5^ mbar. This allows for in situ thermal sample activation and pure gas atmospheres. X‐band EPR measurements were performed on a Bruker EMXplus‐10/12/P/L spectrometer equipped with an EMX^Plus^ standard cavity. Spectra acquisition was performed by the use of a finger Dewar at liquid nitrogen temperatures (*T=*77 K) under dynamic high vacuum conditions (*p*(O_2_)<10^−5^ mbar). At a microwave frequency of 9.35 GHz for EPR measurements, typical acquisition parameters correspond to a field modulation frequency of 100 kHz. Typically, a modulation amplitude of 0.1 mT and microwave powers of 1 mW were selected for spectrum acquisition.

**Computational details**: For modeling the Co/MgO system in its local minimum structure, we used a cubic cell of 6x6x6 ions with the stoichiometry of either Mg_108_O_108_ or CoMg_107_O_108_ and the lattice constant of 421.2 pm experimentally obtained for MgO.^[17]^ The PBE functional, the projector‐augmented wave method (PAW),^[18]^ D2 dispersion correction,^[19]^ periodic boundary conditions and the energy cut‐off of 600 eV were applied, with only the Gamma point in the reciprocal space. Out of this cell, clusters were cut and used for photochemical calculations. For molecular dynamics, we employed a smaller 4x4x4‐ion cubic system of CoMg_31_O_32_ stoichiometry. The system was propagated for 180 ps with the time step of 2 fs and temperature of 300 K kept by the Nose‐Hoover thermostat, a lower energy cut‐off of 300 eV was applied. Initial 40 ps were considered as thermalization period, structures for spectra modeling were cut from the remaining trajectory. For calculations of MgO(001) surfaces, the same cells were used, with the spacing of at least 15 Å between periodic images in the *z* direction and half of layers kept frozen during optimization or molecular dynamics. Adsorbed $O_{2}^{-}$ was optimized on the Mg_108_O_108_(001) surface.

There are various approaches to model electronic states in solids and on surfaces present in the literature, reaching from the direct Δ‐SCF approach and time‐dependent density functional theory (TDDFT) simulations to multi‐reference methods.^[20]^ Here, we combine the multi‐reference calculations for Co^2+^ embedded in MgO modelled by point charges with TDDFT calculations for adsorbed $O_{2}^{-}$ . Note that within the charge embedding scheme, no effective core potentials (ECP) were used for cations surrounding the cluster described on the quantum chemical level, possibly leading to artificial polarization for the anions on the cluster border.

Photochemical calculations on solids that include Co^2+^ were performed using the Multireference Configuration Interaction (MRCI) method. For calculations in minima, an active space of 15 electrons in 10 orbitals, (15,10), including cobalt 3s, 3*p*, 3*d*, 4s orbitals was used. The smallest reasonable active space, (7,5) covering 3*d* orbitals, was used for spectra modeling. 28 doublet and 10 quartet electronic states were considered. Spin‐orbit coupling was included using the Breit‐Pauli operator within the state interacting method. The basis set has a pronounced effect on the excitation energies, with the shift from aug‐cc‐pVDZ to aug‐cc‐pVQZ reducing the average error in excitation energies of the gas‐phase Co^2+^ ion from 0.26 to 0.18 eV for MRCI(7,5); the aug‐cc‐pVQZ basis set is thus used. In Co/MgO calculations, CoO_6_^10−^ and CoO_5_^8−^ were used as the core clusters treated explicitly at the MRCI level for bulk and surface calculations, respectively, with surrounding Mg^2+^ and O^2−^ ions modeled as point charges of +/−1.6 |e|, with the field of Mg_62_O_56_ and Mg_37_O_32_ for bulk and surface structures, respectively; cc‐pVDZ basis set is used for oxygen atoms. For the MRCI approach, all but 7 valence Co electrons were cored to speed up the calculations. Jahn–Teller distortion is not accounted for in the local minimum structure. Absorption spectra were modeled using the reflection principle^[21]^ through calculation of excitation energies in 140 snapshots taken from the molecular dynamics trajectory, the electronic transitions were convoluted by Gaussian functions with the width of 0.02 eV.

Electronic transitions in $O_{2}^{-}$ MgO(001) were calculated using the TDDFT approach with the CAMB3LYP functional and the aug‐cc‐pVTZ basis set for $O_{2}^{-}$ , cc‐pVDZ for the Mg_24_O_24_ cluster next to the adsorbed $O_{2}^{-}\;$ and point charges of +/−1.6 |e| for further Mg_136_O_136_ ions in the vicinity.

Calculations of periodic systems were performed in the VASP program, version 5.4.4,^[22]^ MRCI calculations in Molpro^[23]^ and TDDFT calculations in Gaussian.^[24]^

## Results and Discussion

**Figure 1:**
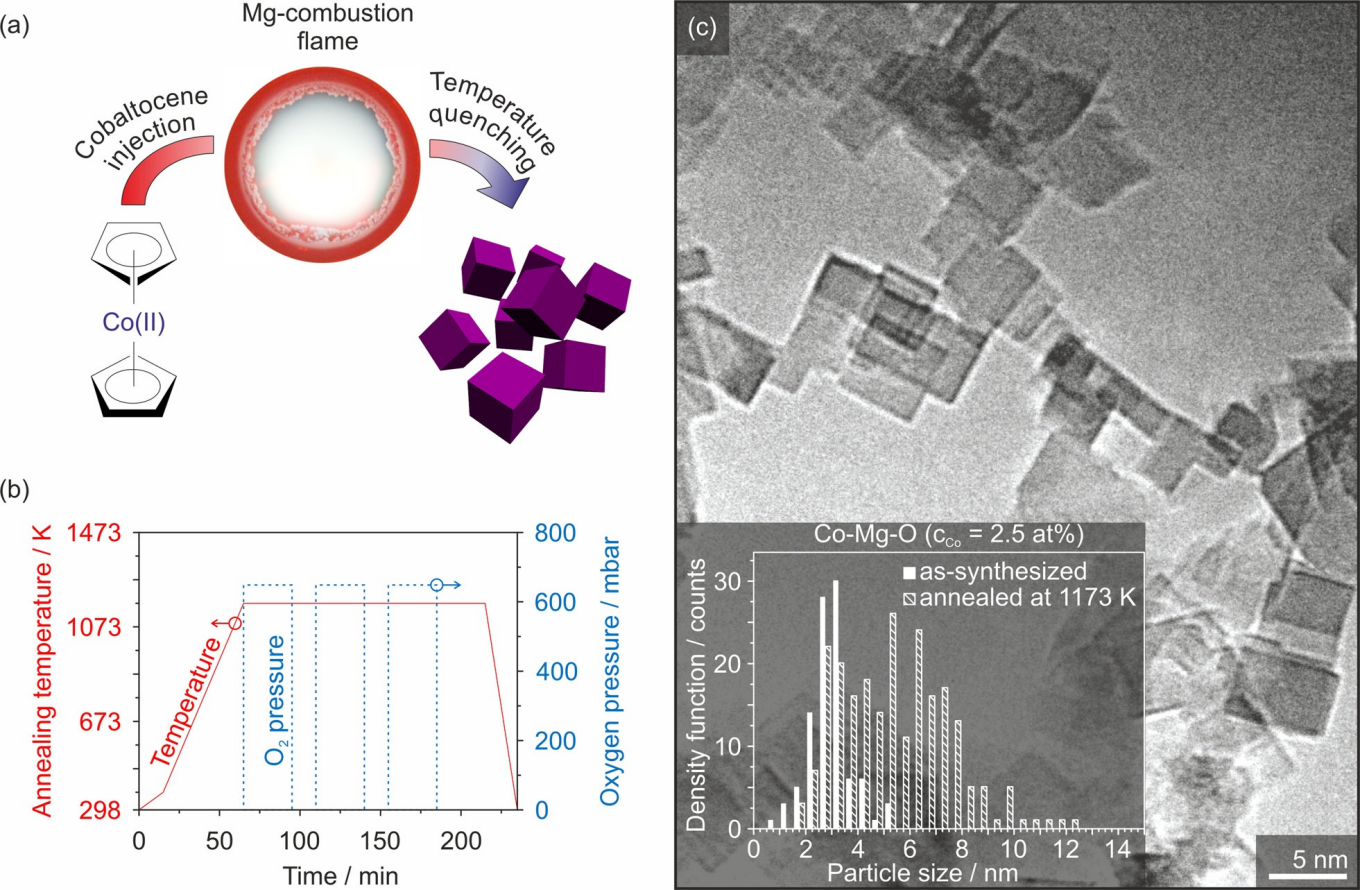
Schematic illustration of cobaltocene decomposition in the Mg combustion flame as particle synthesis approach (a) followed by temperature quenching of the nanoparticle powders with subsequent thermal treatment up to *T=*1173 K according to an optimized process (b). The particle size distributions of the as‐synthesized and 1173 K annealed Co‐Mg‐O nanoparticles are plotted as insets in the TEM bright field image in (c) which also evidences the pronounced cubic shape.

The production of transition metal oxide doped MgO nanocubes in the gas phase utilizes the decomposition of cobaltocene inside the strongly exothermic Mg combustion flame (Figure 1 a). Such an approach provides high control over the integral concentration and homogeneous distribution of the admixed cobalt ions over the metal oxide nanoparticles.^[6]^ A dedicated annealing protocol with vacuum annealing at base pressures as low as *p*(O_2_)<10^−6^ mbar (Figure 1 b) was developed for nanoparticle purification, that is, for the generation of particles with surfaces that are exempt from synthesis related carbonaceous species, adsorbed water or hydroxyls as remnants. As a result of annealing, the particle size distribution adopts slightly larger values, as reflected by the shift of the median value from 3 to 5 nm (Figure 1 c). The high level of stability with regard to particle coarsening is perfectly consistent with the behavior of undoped MgO nanocubes. This observation indicates that the Co^2+^ impurity admixture does not promote particle coarsening and the reduction of specific surface area.

Only minor change in the local MgO structure upon introduction of Co^2+^ ions is also predicted by our calculations. When the Co^2+^ ion is introduced into the MgO structure, the local structure remains almost unaffected, with the bond length ratio *r*(Co‐O)/*r*(Mg‐O) of 1.013. As expected from the gas phase configuration, the high‐spin (quartet) state of Co^2+^ is more stable than the low‐spin doublet state. We also found that the exchange of Mg^2+^ ions against Co^2+^ ions does not affect the surface energy that was calculated to be 1.30 J m^−2^ and 1.29 J m^−2^ for MgO(001) and MgO(001) with one Co^2+^ ion residing on the surface, respectively. (Experimentally, values in the range between 1.04 and 1.20 J m^−2^ were obtained for MgO(001).^[25]^) Our results of a recent X‐ray spectroscopy study on these nanoparticle powders revealed that the Co^2+^ ions in MgO remain within the highly symmetric octahedral environment and do not change their (+2) valence state and are perfectly in line with the here presented results. A high definition of the cubic particle habit with a high abundance of sharp edges and corners is retained after annealing at 1173 K. Thus, annealing in vacuum and oxygen‐rich environment does not affect the hierarchy of surface energies and Co‐doped MgO(100) surfaces remain significantly lower in energy as compared to all other crystal planes.^[6]^

**Figure 2:**
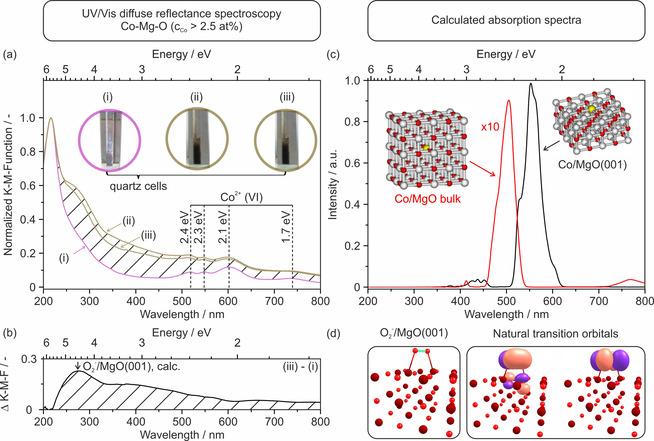
UV/Vis diffuse reflectance spectra (a, top left) of Co‐Mg‐O nanocube powders (i) after vacuum annealing to 1173 K, (ii) after O_2_ addition (*p*(O_2_)=100 mbar) at room temperature and (iii) subsequent evacuation. The difference spectrum (b, bottom left) shows the change in the optical powder properties between (i) the situation prior to oxygen admission and (iii) after subsequent evacuation. The arrow in (b) marks the position of the first bright transition of $O_{2}^{-}$ calculated at the TDDFT level for the model shown in (d). Calculated MRCI absorption spectra of Co^2+^ in the bulk and on the MgO(001) surface are shown in (c). Structure of $O_{2}^{-}$ /MgO(001) used for calculation of excited states and natural transition orbitals of the first bright transition are shown in (d).

Diffuse reflectance spectroscopy measurements in the UV/Vis range (Figure 2, left) were performed on annealed Co‐Mg‐O (*c*_Co_>2.5 at %) powders under high vacuum (*p*(O_2_) <10^−5^ mbar) conditions (Figure 2 a (i, iii)) and in the presence of oxygen (*p*(O_2_)=100 mbar, Figure 2 a (ii)). Independent of the type of surrounding gas atmosphere, all powder samples investigated show a maximum in absorption at 218± 2 nm, which is attributed to exciton formation at the MgO surface. The underlying excitation process involves 4‐fold coordinated O^2−^ anions located on cube edges of the MgO particle. Different from spectra acquired on nanoparticle powders of undoped MgO, several absorption bands in the visible range are measured for the Co‐Mg‐O nanoparticle powders. While related bands do not change their intensity upon oxygen admission, a broad absorption signal emerges in the range between 250 and 800 nm (see difference spectrum, Figure 2 b). Subsequent sample evacuation down to *p*(O_2_) <10^−5^ mbar leads to a slight intensity decrease of this broad absorption feature ((ii)→(iii)). The oxygen adsorption induced absorption features ((iii)–(i)) show a relative maximum roughly at 300 nm that is superimposed on the broad signal ranging up to 800 nm. The observation that this absorption is almost stable after subsequent evacuation points to the presence of at least two different types of surface Co^2+^ ions that enable reversible and irreversible oxygen binding, as suggested by Giamello and Sojka who investigated corresponding high surface area materials that were obtained by the thermal decomposition of hydroxides.^[14b]^

The characteristic pink color of the Co‐doped MgO nanocube powders is associated with Co^2+^‐specific absorption bands[^14c^, ^26^] in the wavelength range between 500 and 800 nm of the UV/Vis diffuse reflectance spectrum (Figure 2 a). The band positions are consistent with electronic transitions within the d^7^ configuration of Co^2+^ ions that substitute Mg^2+^ sites with octahedral local symmetry.^[27]^ Band maxima at 2.1 eV (≈16 900 cm^−1^), 2.3 eV (≈18 600 cm^−1^) and 2.4 eV (≈19 300 cm^−1^) (Figure 2 a) have been attributed to Co^2+^ transitions from the Γ_4_ (^4^F) ground state to the Γ_4_ (^4^P) level.^[27a]^ While number and relative intensity of the bands differ from sample to sample and depend on the host matrix around the octahedrally coordinated Co^2+^ ions,^[28]^ the here measured absorption features of the Co^2+^‐doped MgO nanocrystal powder are in very good agreement with absorption bands measured for Co‐doped single‐crystals in the transmission mode.

Oxygen admission leads to both the emergence of a broad absorption feature between 250 up to 800 nm and an instantaneous change in the color from cobalt pink to dark brown. Whereas the intensity of related absorption feature slightly decreases upon subsequent evacuation to high vacuum, the dark brownish coloration remains unchanged.

The broad absorption feature between 300 and 600 nm can be rationalized by oxygen radical formation that occurs upon interfacial charge transfer from the particle to surface adsorbed molecular oxygen.^[29]^ The XAS analysis of Co‐Mg‐O nanocubes suggests that the majority of Co^2+^ chromophores is located in the particles bulk where they cannot participate in interfacial charge transfer steps. Hence, the electrons that become scavenged by adsorbed oxygen molecules must originate from the surface Co^2+^ ions with a local environment of lower symmetry and, thus, less distinct optical absorption features.

The nature of Co^2+^ electronic transitions in MgO is analyzed using quantum photochemical calculations. In the energy range below 3 eV, there are 15 electronic states for Co^2+^ ions in the gas phase, followed by a gap of about 1.6 eV,^[30]^ all sharing the 3*d* ^7^ electronic configuration. Accounting for degeneracies and spin‐orbit coupling, 96 electronic states are retrieved. The MRCI(7,5) approach is able to reproduce the gas phase experiments with the average error of 0.18 eV and the description is significantly improved with the larger active space, with the average error of MRCI(15,10) of 0.06 eV (see Table S1 in the Supporting Information). The spin‐orbit splitting is well reproduced by our calculations.

The interaction of the Co^2+^ ion with the MgO lattice was modeled through CoO_6_^10−^ and CoO_5_^8−^ clusters placed in the field of magnesium and oxygen ions represented by point charges. When a Co^2+^ ion substitutes a Mg^2+^ ion in the bulk MgO structure or on the MgO(001) surface, considered excitation energies are shifted up by the average energy of 0.44 and 0.33 eV, respectively. Table 1 shows electronic states of Co^2+^ in the octahedral field of the MgO lattice.

**Table 1:** Energy of electronic states (in eV) of Co^2+^ incorporated in the MgO lattice as the CoO_6_^10−^ cluster embedded in point charges with and without accounting for spin‐orbit coupling (SO) along with state degeneracy in parenthesis. The MRCI(7,5) method was used with aug‐cc‐pVQZ and aug‐cc‐pVDZ basis sets for Co and O, respectively.

State	MRCI	MRCI+SO
^4^T_1*g*_ (^4^F)	0.00	0.00 (2); 0.04 (4); 0.12 (4); 0.13 (2)
^4^T_2*g*_ (^4^F)	0.69	0.76 (2); 0.77 (4); 0.78 (4); 0.81 (2)
^4^A_2*g*_ (^4^F)	1.51	1.60 (4)
^4^T_1*g*_ (^4^P)	2.42	2.46 (2); 2.48 (4); 2.50 (4); 2.55 (2)
^2^E_*g*_ (^2^G)	1.77	1.85 (4)
^2^T_2*g*_ (^2^G)	2.37	2.41 (4); 2.47 (4); 2.50 (2); 2.51 (2)
^2^T_1*g*_ (^2^G)	2.38	
^2^A_1*g*_ (^2^G)	2.86	2.93 (2)
^2^T_1*g*_ (^2^P)	2.93	3.01 (4); 3.09 (2)
^2^T_2*g*_ (^2^H)	3.30	3.37 (2); 3.41 (4)
^2^T_1*g*_ (^2^H)	3.42	3.49 (2); 3.50 (4)
^2^E_*g*_ (^2^H)	3.59	3.69 (4)
^2^T_1*g*_ (^2^H)	4.02	4.10 (2); 4.15 (4)
^2^T_2*g*_ (^2^D)	3.89	3.95 (2); 3.96 (4)
^2^E_*g*_ (^2^D)	3.95	4.06 (4)

All transitions between the ground electronic state and excited states are symmetrically forbidden for isolated Co^2+^ ions in the gas phase. The same situation applies to the Co^2+^ ion in the octahedral field of Mg^2+^/O^2−^ ions, with all excitations having zero transition dipole moments. For the surface structure, however, the asymmetric field induces non‐zero transition probabilities. The experimentally measured band intensities thus arise from the non‐symmetrical local structures that result from MgO lattice vibrations; for Co^2+^ on the surface or as part of defect structures where they are coordinatively unsaturated, the asymmetric field further contributes to the intensity increase.

To model the absorption spectrum of Co^2+^/MgO, we sampled the system using molecular dynamics and constructed the spectrum by calculating excitation energies in 140 snapshots along the molecular dynamics run (note that molecular dynamics is used to retrieve the ground state density, not to follow system evolution in time). The spectrum obtained is shown in Figure 2 c for a Co^2+^ ion either in the particle bulk or as part of the MgO(001) surface.

Transitions into the electronic states in the 2.1–2.7 eV region gain considerably in intensity while the intensities of other transitions in the 1.5‐4 eV range are at least by one order of magnitude lower. For Co^2+^ on the MgO(001) surface, the relevant absorptions are shifted by about −0.2 eV with respect to bulk Co^2+^, further shifts can be expected for defect sites. Due to the asymmetric field, the Co^2+^ ions on the surface are predicted to absorb with intensities that are by factor of 10 higher as compared to Co^2+^ in the bulk.

Within the limits of our computational approach, the calculations match well to the experimental observations. The bands observed in the experiment could be assigned as ^4^A_2*g*_ (^4^F) (the low‐energy band at 1.7 eV), ^4^T_1*g*_ (^4^P) and ^2^T_2*g*_/^2^T_1*g*_ (^2^G) states (for the 2.1–2.7 eV region) for Co^2+^ in bulk. Thus, also spin‐forbidden transitions can be observed when we account for spin‐orbit effects, they are responsible for about 20 % of spectral intensity shown in Figure 2 c for Co^2+^ in bulk MgO. For Co^2+^ on the surface, the ^4^E, ^4^A_2_ (^4^P) and doublet states correlating to the ^2^G state of the gas‐phase Co^2+^ ion contribute at 2.1–2.4 eV. The experimentally observed shift to higher excitation energies between Co^2+^ in the gas phase and incorporated in MgO agrees with the calculated shift of about +0.3 eV. The analysis of the spectral shape is complicated by the fact that surface and defect structures are expected to have higher transition dipole moments compared to Co^2+^ in the bulk, that is, structures of lower probability have a relatively higher weight in the absorption spectrum. However, our calculations explain why there are no bands observed in the 2.7–4.0 eV region. Although several Co^2+^ excited states are present in this area, they do not gain sufficient intensity through lattice vibrations and thus do not appear in the spectrum. According to the calculations the experimentally observed band at 1.7 eV must originate from the Co^2+^ ion in the bulk corresponding to the ^4^A_2*g*_ (^4^F) target state, while the analogous transition for the Co^2+^ ion on the MgO(001) surface into the ^4^B_1_ (^4^F) state lies at 1.25 eV. However, in principle this band could arise also due to some specific surface structures. (Note that this band was reported in the study of ref. [26] but not in ref. [27a].)

**Figure 3:**
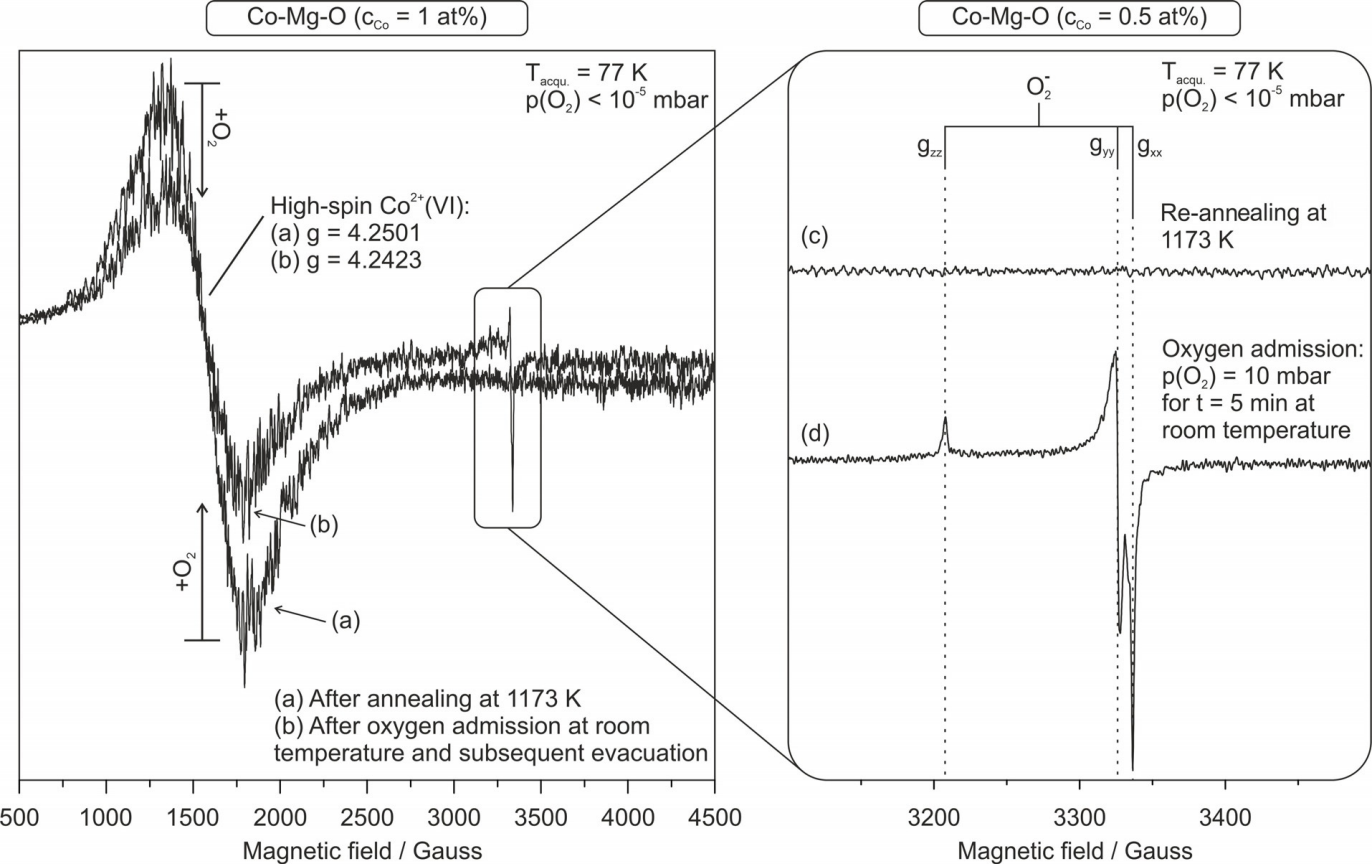
Left panel: wide range magnetic field EPR spectrum (left) of 1173 K annealed Co‐Mg‐O (*c*_Co_=1 at %) before (a) and after oxygen addition (b) and, in the right panel: the magnified fingerprint region of superoxide anions on Co‐Mg‐O (*c*_Co_=0.5 at %), after (c) in situ re‐annealing up to 1173 K and (d) oxygen addition at room temperature.

Investigations of potential paramagnetic states that form upon O_2_ adsorption were performed with X‐band electron paramagnetic resonance (EPR) spectroscopy. Figure 3 shows EPR spectra of annealed Co‐Mg‐O samples with different concentrations and representative for all investigated samples. Spectra were acquired prior to (Figure 3 a, c) and after (Figure 3 b, d) oxygen admission (*p*(O_2_)=10 mbar) under dynamic high vacuum conditions (*p*(O_2_)<10^−5^ mbar) at liquid nitrogen temperatures (*T*_acqu._=77 K). Wide range magnetic field measurements (Figure 3 a, b) down to magnetic fields as low as 500 G were performed in order to capture the resonance of the transition metal ion cobalt. An extremely broad, isotropic signal of about 2300 G width and an average *g*‐value of *g=*4.250 before (Figure 3 a) and *g=*4.242 after oxygen admission (Figure 3 b) can be detected. The signal is characteristic for high‐spin bulk Co^2+^ ions in the octahedral environment of MgO.[^14a^, ^14c^, ^14d^] In addition to the slight shift in the *g*_average_ value we also observe a slight decrease in the Co^2+^ signal intensity. An additional resonance related to a spin center of orthorhombic local symmetry emerges at higher magnetic field values and results from local charge transfer reactions between the nanocrystals and adsorbed oxygen.^[14b]^ Sample annealing to 1173 K leads to an extinction of the paramagnetic signal (Figure 3 c), subsequent O_2_ addition at room temperature however regenerates the EPR signal (Figure 3 d). Note that such interfacial charge transfer reactions do not occur on undoped MgO nanostructures and independent of the way of their production.[^14a^, ^14c^, ^14d^, ^29^, ^31^] The complex three‐component signal, with diagonal *g*‐tensor values of *g_zz_*=2.0820, *g_yy_*=2.0081 and *g_xx_*=2.0017 is consistent with side‐on bound superoxide anions ($O_{2}^{-}$ ) that are stabilized at Mg^2+^ ions in edge positions of the Co‐Mg‐O nanocube surface.[^14d^, ^31^] Interestingly, we do not obtain evidence for the presence of $Co^{3+}{-O}_{2}^{-}$ adsorption complexes although the electrons must originate from a small fraction of surface Co^2+^ ions which must become oxidized to Co^3+^ in the course of this interfacial electron transfer step. This may be due to the formation of diamagnetic low‐spin Co^3+^ complexes that do not contribute to the EPR resonance signal. Our calculations on the small 6x6x6 periodic Co/MgO model predict that adsorption of $O_{2}^{-}$ on the cobalt center is considerably more favorable compared to the conformation when it is coordinated to Mg^2+^ ions with the surface cobalt ion separated from it by one more Mg^2+^ ion. In the structure with direct Co‐O_2_ interaction, the cobalt ion is calculated to adopt the low‐spin state, in line with the experimental data (see the SI for further details and analysis of the computational model limitations). However, high‐level ab initio methods would be needed to confirm the DFT/PBE predictions.

Moreover, one can also see that the integral intensity related to the signal of the paramagnetic oxygen species is by far smaller than the O_2_‐admission intensity change of the Co^2+^(VI) resonance in the low magnetic field region. This suggests that under the present conditions, only a fraction of anionic oxygen species is in fact EPR active and the majority of adsorbed oxygen species is diamagnetic in nature and forms peroxides and decomposition products thereof.

The complementary study with optical absorption and electron paramagnetic resonance spectroscopy reveals that the change in the optical absorption is associated with an interfacial charge transfer from Co^2+^ to surface adsorbed oxygen. The observation of the instantaneous color change from cobalt pink to a dark brown (Figure 2 a (i, ii)) is accompanied by the emergence of a three‐component paramagnetic signal as an EPR spectroscopic fingerprint of superoxide anions ($O_{2}^{-}$ ) stabilized on the MgO surface. Thus, the emergent optical absorption feature in the range between 250 and 800 nm provides evidence for an efficient d‐electron transfer from Co^2+^ located at the particle surface to molecular oxygen. The optical fingerprint corresponds to the absorption of adsorbed anionic oxygen species that become stabilized on the MgO surface and is superimposed on Co^2+^ related absorption features. A fraction of these oxygen species is paramagnetic and corresponds to superoxide anions ($O_{2}^{-}$ ). The evidence for the latter species is further supported by our TDDFT calculations of $O_{2}^{-}$ absorbed on the MgO(001) surface that predict the first bright excitation of $O_{2}^{-}$ to lie at 4.6 eV, practically at the same position as the maximum of the experimentally measured band (see Figure 2 b). Again, the here reported spectroscopic property changes (Figures 2 and 3) were exclusively observed on Co‐Mg‐O nanostructures, but not for samples of pure and undoped MgO nanoparticles.[^14a^, ^14c^, ^14d^, ^29^, ^31^]

## Conclusions

In this combined experimental and theoretical study, we addressed for the first time reactivity and spectroscopic fingerprints of isolated Co^2+^ ions dispersed over the bulk and surface of unsupported MgO nanocubes. We assigned measured bands in the range of visible light to characteristic electronic transitions of Co^2+^ ions either in octahedral coordination environment (in the bulk) or those being part of surface or defect structures. There the probability of these originally symmetry‐forbidden transitions and, thus, the intensity is increased due to lattice vibrations or the presence of an asymmetrical ion field.

At room temperature, the redox chemistry of vacuum annealed and Co‐doped MgO nanocubes has been monitored by changes in optical absorption properties and—at the same time—the formation of paramagnetic surface oxygen radicals. The high level of agreement between experiment and theory in combination with the extreme uniformity in Co^2+^ dispersion and particle properties^[6]^ makes related metal oxide nanoparticle systems a promising candidate for sensor applications, for model studies in heterogeneous catalysis and in particular for atomic level studies on single atomic catalysts.^[32]^ Depending on the location of the Co^2+^ admixtures, particle bulk or surface, the composition of the surrounding gas atmosphere can be used to selectively tune optical powder properties leading to absorption bands with energies that are consistent with accessible energy states from ab‐initio calculations. The here applied approach of controlling dispersion of extrinsic defects and their redox activity opens pathways towards surface functionalization of oxide nanomaterials with single ionic sites. It represents a substantial advance in the rationalization and subsequent control over dispersion and reactivity of extrinsic point defects in response to chemical changes at room temperature. In addition to the above‐mentioned potential for single site catalysts, such particle powders are extremely well‐defined precursor materials for functional ceramics with chemically tuned interfaces.

## Conflict of interest

The authors declare no conflict of interest.

## Supporting information

As a service to our authors and readers, this journal provides supporting information supplied by the authors. Such materials are peer reviewed and may be re‐organized for online delivery, but are not copy‐edited or typeset. Technical support issues arising from supporting information (other than missing files) should be addressed to the authors.

Supplementary
