# Supplementary material for: Isolated Cobalt Ions Embedded in Magnesium Oxide Nanostructures: Spectroscopic Properties and Redox Activity
Source: Chemistry. 2020 Oct 19;26(68):16049–56. doi: 10.1002/chem.202002817 (PMC7756418; doi:10.1002/chem.202002817)
Supplement: Supplementary file 1 — Supplementary [file CHEM-26-16049-s001.pdf]

Chemistry–A European Journal

Supporting Information

Isolated Cobalt Ions Embedded in Magnesium Oxide Nanostructures: Spectroscopic Properties and Redox Activity

Thomas Schwab,[a] Matthias Niedermaier,[a] Gregor A. Zickler,[a] Milan Ončák,*[b] and Oliver Diwald*[a]

Supporting Information

Additional computational data

Calculations of O_2^- adsorption

Figure S1 shows three models considered for modeling of O_2^- adsorption – MgO(001) surface and two different positions on the Co/MgO(001) surface. For the adsorption on the MgO(001), configuration **I**, we observe a very strong interaction with the Mg^{2+} cations, with the Mg-O distance of 2.17 Å, the O-O distance is 1.36 Å.

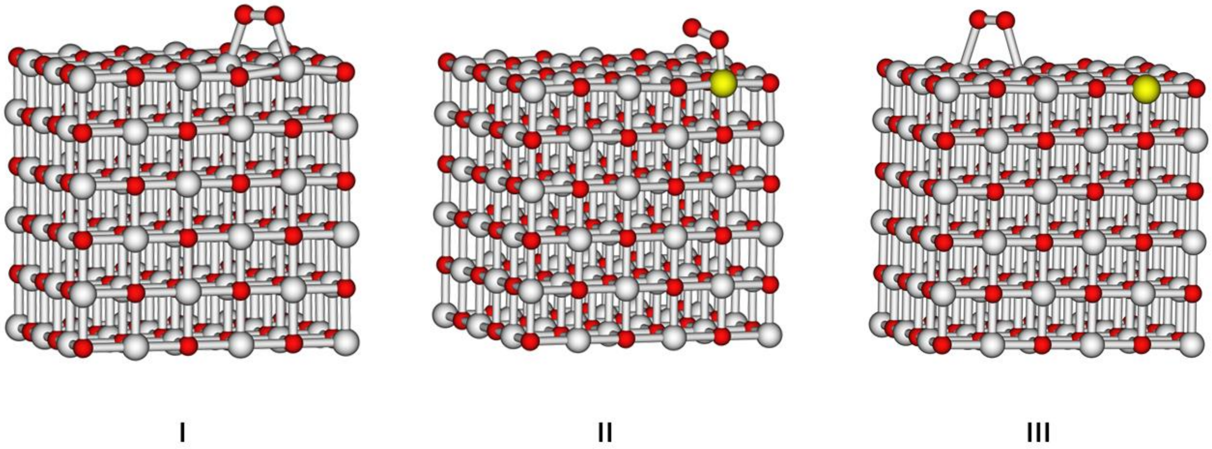

Figure S1: Three structures considered for O_2^- adsorption on the MgO(001) and Co/MgO(001) surface. Optimized at the PBE+D level, cut-off energy of 600 eV. See the Methods section of the manuscript for further technical details. Oxygen – red, magnesium – white, cobalt – yellow.

For calculation of O_2^- adsorption on the Co/MgO(001) surface, direct adsorption on the Co center (configuration **II** in **Figure S1**) is predicted to possess the lowest spin state (quartet is by 0.34 eV less stable than the doublet state). Here, the O-O distance is 1.32 Å, the interaction with the cobalt ion is very strong, with the Co-O distance of 1.81 Å. For the corresponding structure with O_2^- adsorbed on Mg atoms in the high-spin state, configuration **III**, positioned one Mg away from the cobalt center, the O-O bond is stronger with 1.28 Å, the Mg-O distances are 2.49 and 2.52 Å. This

shows that the O_2 moiety cannot be clearly assigned as O_2^-, with spin density of 0.77 on both oxygen atoms as calculated through Bader analysis. This configuration lies by 0.90 eV higher compared to the configuration **II** with direct O_2-Co interaction. The striking structural difference between structures **I** and **III** shows the limits of the computational model with the 6x6 surface and the cobalt ion positioned next to the adsorbed O_2^-. The cobalt ion is not far enough and influences the binding considerably. Thus, the calculated energy difference should be taken with caution. At the same time, we note that the DFT/PBE method might fail to assign the correct spin multiplicity of the ground electronic state and, although the low-spin state of configuration **II** is consistent with the experimental observations, higher-level calculations should be conducted to confirm the results.

Benchmarks and excitation energies in bulk and surface

Table S1: Energy of electronic states (in eV) of Co^{2+} in the gas phase using the aug-cc-pVQZ basis set. Experimental values are taken from Kramida, A, Ralchenko, Yu, Reader, J. and NIST ASD Team (2019). NIST Atomic Spectra Database (version 5.7.1). Available: https://physics.nist.gov/asd [Wed Apr 08 2020]. National Institute of Standards and Technology, Gaithersburg, MD.

State	Co^{2+}, gas phase		
	Experiment	MRCI(7,5)	MRCI(15,10)
$^4F_{9/2}$	0.00	0.00	0.00
$^4F_{7/2}$	0.10	0.11	0.11
$^4F_{5/2}$	0.18	0.19	0.19
$^4F_{3/2}$	0.23	0.24	0.25
$^4P_{5/2}$	1.88	2.01	1.95
$^4P_{3/2}$	1.91	2.05	1.98
$^4P_{1/2}$	1.96	2.09	2.03
$^2G_{9/2}$	2.11	2.30	2.17
$^2G_{7/2}$	2.20	2.40	2.28
$^2P_{3/2}$	2.50	2.87	2.54
$^2P_{1/2}$	2.59	2.99	2.64
$^2H_{11/2}$	2.82	3.06	2.94

$^2H_{9/2}$	2.91	3.15	3.03
$^2D_{5/2}$	2.86	3.10	2.92
$^2D_{3/2}$	3.01	3.27	3.07

Table S2 – Energies of electronic states (in eV) for bulk and surface models of Co/MgO systems. Calculated at the MRCI(7,5)/aug-cc-pVQZ(Co),aug-cc-pVDZ(O) level of theory, spin-orbit coupling is included.

CoO_6^- core, bulk	CoO_5^- core, surface
0.00	0.00
0.00	0.00
0.04	0.03
0.04	0.03
0.04	0.10
0.04	0.10
0.12	0.11
0.12	0.11
0.12	0.15
0.12	0.15
0.13	0.15
0.13	0.15
0.76	0.35
0.76	0.35
0.77	0.37
0.77	0.37
0.77	0.70
0.77	0.70
0.78	0.72
0.78	0.72
0.78	0.75
0.78	0.75
0.81	0.78
0.81	0.78
1.60	1.25
1.60	1.25
1.60	1.25
1.60	1.25
1.85	1.70
1.85	1.70
1.85	2.09
1.85	2.09
2.41	2.15
2.41	2.15
2.41	2.19
2.41	2.19
2.46	2.20
2.46	2.20
2.47	2.21
2.47	2.21
2.47	2.23
2.47	2.23
2.48	2.33
2.48	2.33
2.48	2.35
2.48	2.35
2.50	2.46
2.50	2.46
2.50	2.55
2.50	2.55
2.50	2.56
2.50	2.56
2.51	2.73
2.51	2.73

2.55	2.76
2.55	2.76
2.93	2.84
2.93	2.84
3.01	2.90
3.01	2.90
3.01	2.91
3.01	2.91
3.09	2.92
3.09	2.92
3.37	3.24
3.37	3.24
3.41	3.28
3.41	3.28
3.41	3.31
3.41	3.31
3.49	3.45
3.49	3.45
3.50	3.47
3.50	3.47
3.50	3.55
3.50	3.55
3.69	3.56
3.69	3.56
3.69	3.64
3.69	3.64
3.95	3.65
3.95	3.65
3.96	3.73
3.96	3.73
3.96	3.77
3.96	3.77
4.06	3.78
4.06	3.78
4.06	3.79
4.06	3.79
4.10	3.84
4.10	3.84
4.15	3.90
4.15	3.90
4.15	4.00
4.15	4.00

Cartesian coordinates of minima (in Ångstrom)

Co/MgO, bulk
Co 0.0 0.0 0.0
O 0.0 0.0 -2.13266
O 0.0 -2.13266 0.0
O -2.13266 0.0 0.0
O 0.0 0.0 2.13266
O 0.0 2.13266 0.0
O 2.13266 0.0 0.0
Mg 0.0 -2.10659 -2.10659
Mg -2.10659 0.0 -2.10659
Mg -2.10659 -2.10659 0.0
Mg 0.0 2.10659 -2.10659
Mg 0.0 -2.10659 2.10659
Mg 2.10659 0.0 -2.10659
Mg 2.10659 -2.10659 0.0
Mg -2.10659 0.0 2.10659
Mg -2.10659 2.10659 0.0
Mg 0.0 2.10659 2.10659
Mg 2.10659 0.0 2.10659
Mg 2.10659 2.10659 0.0
O -2.10524 -2.10524 -2.10524
O 2.10524 -2.10524 -2.10524
O -2.10524 2.10524 -2.10524
O -2.10524 -2.10524 2.10524
O 2.10524 2.10524 -2.10524
O 2.10524 -2.10524 2.10524
O -2.10524 2.10524 2.10524
O 2.10524 2.10524 2.10524
Mg 0.0 0.0 4.22175
Mg 0.0 0.0 -4.22175
Mg 0.0 4.22175 0.0
Mg 0.0 -4.22175 0.0
Mg 4.22175 0.0 0.0
Mg -4.22175 0.0 0.0
O 0.0 2.10712 -4.2138
O 0.0 -4.2138 2.10712
O 0.0 -4.2138 -2.10712
O 0.0 -2.10712 -4.2138
O 2.10712 0.0 -4.2138
O 2.10712 -4.2138 0.0
O -4.2138 0.0 2.10712
O -4.2138 0.0 -2.10712
O -4.2138 2.10712 0.0
O -4.2138 -2.10712 0.0
O -2.10712 0.0 -4.2138
O -2.10712 -4.2138 0.0
O 0.0 2.10712 4.2138
O 0.0 4.2138 2.10712
O 0.0 4.2138 -2.10712
O 0.0 -2.10712 4.2138
O 2.10712 0.0 4.2138
O 2.10712 4.2138 0.0
O 4.2138 0.0 2.10712
O 4.2138 0.0 -2.10712
O 4.2138 2.10712 0.0

O 4.2138 -2.10712 0.0
O -2.10712 0.0 4.2138
O -2.10712 4.2138 0.0
Mg 2.1059 2.1059 4.21195
Mg 2.1059 2.1059 -4.21195
Mg 2.1059 4.21195 2.1059
Mg 2.1059 4.21195 -2.1059
Mg 2.1059 -4.21195 2.1059
Mg 2.1059 -4.21195 -2.1059
Mg 2.1059 -2.1059 4.21195
Mg 2.1059 -2.1059 -4.21195
Mg 4.21195 2.1059 2.1059
Mg 4.21195 2.1059 -2.1059
Mg 4.21195 -2.1059 2.1059
Mg 4.21195 -2.1059 -2.1059
Mg -4.21195 2.1059 2.1059
Mg -4.21195 2.1059 -2.1059
Mg -4.21195 -2.1059 2.1059
Mg -4.21195 -2.1059 -2.1059
Mg -2.1059 2.1059 4.21195
Mg -2.1059 2.1059 -4.21195
Mg -2.1059 4.21195 2.1059
Mg -2.1059 4.21195 -2.1059
Mg -2.1059 -4.21195 2.1059
Mg -2.1059 -4.21195 -2.1059
Mg -2.1059 -2.1059 4.21195
Mg -2.1059 -2.1059 -4.21195
Mg 0.0 -4.21239 -4.21239
Mg -4.21239 0.0 -4.21239
Mg -4.21239 -4.21239 0.0
Mg 0.0 4.21239 4.21239
Mg 0.0 4.21239 -4.21239
Mg 0.0 -4.21239 4.21239
Mg 4.21239 0.0 4.21239
Mg 4.21239 0.0 -4.21239
Mg 4.21239 4.21239 0.0
Mg 4.21239 -4.21239 0.0
Mg -4.21239 0.0 4.21239
Mg -4.21239 4.21239 0.0
O 2.10611 4.21195 4.21195
O 2.10611 4.21195 -4.21195
O 2.10611 -4.21195 4.21195
O 2.10611 -4.21195 -4.21195
O 4.21195 2.10611 4.21195
O 4.21195 2.10611 -4.21195
O 4.21195 4.21195 2.10611
O 4.21195 4.21195 -2.10611
O 4.21195 -4.21195 2.10611
O 4.21195 -4.21195 -2.10611
O 4.21195 -2.10611 4.21195
O 4.21195 -2.10611 -4.21195
O -4.21195 2.10611 4.21195
O -4.21195 2.10611 -4.21195
O -4.21195 4.21195 2.10611
O -4.21195 4.21195 -2.10611
O -4.21195 -4.21195 2.10611
O -4.21195 -4.21195 -2.10611

O -4.21195 -2.10611 4.21195
O -4.21195 -2.10611 -4.21195
O -2.10611 4.21195 4.21195
O -2.10611 4.21195 -4.21195
O -2.10611 -4.21195 4.21195
O -2.10611 -4.21195 -4.21195
Mg -4.21194 -4.21194 -4.21194
Mg 4.21194 -4.21194 -4.21194
Mg -4.21194 4.21194 -4.21194
Mg -4.21194 -4.21194 4.21194
Mg 4.21194 4.21194 -4.21194
Mg 4.21194 -4.21194 4.21194
Mg -4.21194 4.21194 4.21194
Mg 4.21194 4.21194 4.21194

Co/MgO, surface
Co 0.0 0.0 0.0
O 2.1074055444 0.00515776248 0.0004428
O -0.00507133224 -2.10744370516 0.0007731
O -2.12521383832 0.0058561542 -0.003273
O -0.00583707384 2.12524302744 -0.0036348
O -0.00979087822 0.00973426896 -2.1634416
Mg 2.10394189044 -2.10396387706 -0.0527604
Mg -0.00873867852 -2.08993993884 -2.126622
Mg 2.08991251872 0.00865363824 -2.1268923
Mg 2.09934769356 2.11695368508 -0.0551238
Mg -2.11693131932 -2.09940670368 -0.0545436
Mg -2.11362612082 2.11363976772 -0.0579528
Mg -2.10472380612 0.0083751408 -2.124777
Mg -0.00844223796 2.10468665628 -2.1250524
O 2.09761934148 -2.09772396756 -2.1323826
O -2.11344087712 -2.09839076928 -2.1329451
O 2.09833630812 2.11339210212 -2.1336147
O -2.11414141692 2.11402188036 -2.1348726
Mg 4.213193049 0.00754230204 -0.0585921
Mg -0.00752132628 -4.21330007596 -0.0583374
Mg -0.00749150532 4.22768793096 -0.059331
Mg -4.2277743612 0.00749390616 -0.059043
Mg -0.00831726792 0.00834077088 -4.2362748
O 4.20500378376 -2.09816243676 0.000450899999999
O 2.09815055892 -4.20515667936 0.000429899999999
O 4.20355632996 2.11230426888 0.0006051
O -2.11230161532 -4.20371819716 0.0011805
O 2.09837181528 4.22054821188 -0.000334500000001
O -4.22065978776 -2.09838369316 0.000191699999998
O -0.00747343584 -4.20346901516 -2.1348591
O 4.20352941528 0.00733671432 -2.1350325
O -2.11255888432 4.21932112992 8.09999999873e-06
O -4.21945317612 2.11255509348 6.89999999999e-05
O -0.00757919912 -2.09921577372 -4.226793
O 2.0992704876 0.00760611384 -4.2268839
O -2.11516038392 0.00758058912 -4.2273555
O -0.0076220352 2.115184266 -4.2274743
O -4.22021449512 0.0073718424 -2.1359127
O -0.00746383248 4.2202047654 -2.1361401
Mg 2.09735840808 -4.20313390846 -2.129973
Mg 4.20314515452 -2.09740301316 -2.12997

Mg -2.11266148862 -4.20286716256 -2.1294135
Mg 4.202904186 2.1126090492 -2.1298503
Mg 2.09739429432 -2.0973681378 -4.22706
Mg -4.21837810524 -2.0972563092 -2.1297171
Mg -2.11297334512 -2.09715433668 -4.2270693
Mg 2.09720968236 4.21842511116 -2.1301599
Mg 2.09720361708 2.11302489996 -4.2272469
Mg -4.21837027092 2.11244882472 -2.1299424
Mg -2.11248117292 4.2183933948 -2.1299625
Mg -2.11283359092 2.11283194824 -4.2272688
Mg 4.20584685768 -4.20595312646 -0.0587286
Mg 4.20481247472 4.22005111164 -0.0586866
Mg -4.22017519716 -4.20492645146 -0.0581754
Mg -0.00765400432 -4.20289534076 -4.2276027
Mg 4.203027387 0.00762885864 -4.2276477
Mg -4.21942310244 4.2193379358 -0.0584472
Mg -4.21805171736 0.00764894988 -4.2274752
Mg -0.00768306708 4.21817112756 -4.2275208
O 4.20399378828 -4.20398608036 -2.1357228
O 2.09847707316 -4.20404534316 -4.2248457
O 4.20415931988 -2.09843499528 -4.2248535
O -2.11353501532 -4.20417460946 -4.2247725
O 4.20429907404 2.1135965526 -4.2249561
O -4.2191869356 -4.20417915836 -2.1355041
O 4.20418876176 4.21925062104 -2.1358743
O -4.21954301808 -2.09869643412 -4.2248625
O 2.09872827684 4.21966103832 -4.2250902
O -4.21979447448 2.11387631364 -4.2251199
O -2.11383234032 4.21991957088 -4.2251115
O -4.21948615608 4.21953935364 -2.1357519
Mg 4.2041861082 -4.20403106446 -4.2280713
Mg -4.21913158992 -4.20405658916 -4.2280353
Mg 4.2041854764 4.21926199344 -4.2281853
Mg -4.21920197244 4.21935853248 -4.2280932

O_2^-/MgO, surface
O 0.0 0.0 0.0
O -0.95830210944 -0.95862142116 -0.000696599999999
Mg 0.53348863464 0.5365946898 -2.0357703
O 0.6197850594 -1.57609358712 -2.3025663
O -1.5792782382 0.62299055988 -2.3018337
Mg -1.49321494764 -1.4901167268 -2.0363544
O 2.69615292336 0.5780793096 -2.201688
O 0.57471952356 2.69955933624 -2.2015536
Mg 2.66704248564 -1.52771615568 -2.2668987
Mg -1.53104371992 2.67044447592 -2.2667574
Mg 0.57145956192 -3.62333205936 -2.2668795
Mg -3.6267163592 0.57484411452 -2.2666614
Mg 2.66562333648 2.6690384682 -2.2573476
O 0.57792426588 0.58128026112 -4.3542396
O -1.5344697186 -3.65265162288 -2.2018125
O -3.6559954876 -1.5310478898 -2.2016331
Mg 0.55641134952 -1.51280567568 -4.3147623
Mg -1.5161424642 0.55973588112 -4.3144629
O -1.5378281147 -1.534462137 -4.3541049
O 2.69447107176 -3.65064565788 -2.2131885
O -3.6540870725 2.69785954152 -2.2128792

Mg 2.65221729864 0.57064277088 -4.3084596
Mg 0.56733264432 2.6555230026 -4.3083909
Mg 4.78015823844 0.57452189652 -2.2636401
Mg 0.57108920076 4.78366081128 -2.2637058
O 2.69711894556 -1.5331303026 -4.3597593
O -1.5365039882 2.70037031472 -4.3595769
O 4.79011856544 -1.53394734636 -2.2099044
O -1.5373677852 4.79353900428 -2.209824
Mg -3.6254518747 -3.62203977564 -2.2571928
O 0.57679940916 -3.65344945988 -4.3597515
O -3.656755922 0.58020986556 -4.3595205
O 2.6815765392 2.68492116204 -4.3441863
Mg -1.52707412052 -3.60871486092 -4.3084785
Mg -3.612055693 -1.52370965916 -4.3083639
O 4.78730200104 2.68776527292 -2.2075059
O 2.684312865 4.7907574416 -2.2074954
O 0.5777924724 -5.74636125958 -2.210091
O -5.74980924492 0.58123691964 -2.2098096
Mg 2.67391065708 -3.6301547412 -4.3325259
Mg -3.6335854152 2.67733943568 -4.33239
Mg -1.53075928356 -5.73649734528 -2.2636446
Mg -5.73998614488 -1.52727654924 -2.2635483
Mg 4.78435111596 -3.634290504 -2.2688622
Mg -3.6377276224 4.78780832556 -2.2687788
Mg 0.57232588608 0.57557978244 -6.4205511
O 4.78594540008 0.57810533976 -4.3473135
O 0.57474694368 4.7892120588 -4.3473729
Mg 4.7836793862 -1.52732519784 -4.3326453
Mg -1.5307016634 4.78700745588 -4.3326096
O 0.57527399124 -1.53158769972 -6.4443588
O -1.5348160714 0.57852763488 -6.444306
Mg 2.67800484744 -5.74061617588 -2.2689255
Mg -5.74407325908 2.68146180432 -2.2687692
O -3.6413695703 -3.63801306958 -4.3441419
Mg -1.53183814524 -1.52859347316 -6.420525
Mg 4.78010491452 2.67791096196 -4.3307847
Mg 2.67456810816 4.78347063948 -4.33083
O 2.68190077896 0.57771956268 -6.4423017
O 0.57453276348 2.6850539664 -6.4422933
Mg 2.67634725696 -1.52691414876 -6.4234935
Mg -1.53014011956 2.67951232224 -6.4234248
Mg 4.78269137736 4.78617133176 -2.2686813
O -3.6441272509 -5.74366511628 -2.2073223
O -5.7471061518 -3.64065753168 -2.2072929
Mg 0.57097269684 -5.74001129048 -4.3327245
Mg -5.74334845812 0.57436129296 -4.3325808
O 6.88619075508 0.58123691964 -2.2098096
O 0.5777924724 6.88963874042 -2.210091
O -1.5344943588 -5.74231445428 -4.3473198
O -5.74558818912 -1.53107644716 -4.3472586
Mg 0.57051956988 -3.63270443328 -6.4235178
Mg -3.6358914852 0.57374174988 -6.4234593
Mg 6.89601385512 -1.52727654924 -2.2635483
Mg -1.53075928356 6.89950265472 -2.2636446
O 4.78914407712 -3.63921033058 -4.3502067
O -3.6426078983 4.79249779788 -4.3501473
Mg 2.67440649372 2.67756701004 -6.4227558

O -1.5339838644 -3.63820374688 -6.4422987
O -3.6413790473 -1.53075524004 -6.4422672
O 2.68286389488 -5.74541355958 -4.3502676
O -5.74879192056 2.68621698384 -4.350183
Mg 6.89192674092 2.68146180432 -2.2687692
Mg 2.67800484744 6.89538382412 -2.2689255
O 4.78907975988 -5.74552374548 -2.2093218
O -5.74897842792 4.7925656532 -2.2092615
O 2.68206264612 -3.63837130018 -6.4433757
O -3.6415979028 2.68528558428 -6.4433448
Mg 4.78022900004 0.57519400536 -6.4240563
Mg 0.57201352416 4.78338774732 -6.4241013
O 4.78546510572 4.7887994934 -4.3492701
Mg -3.6343506514 -5.73646929338 -4.3307376
Mg -5.73983956728 -3.63093615144 -4.330725
O 6.8888938482 -3.64065753168 -2.2072929
O -3.6441272509 6.89233488372 -2.2073223
Mg 6.89265154188 0.57436129296 -4.3325808
Mg 0.57097269684 6.89598870952 -4.3327245
O 4.78926841536 -1.53066969432 -6.4435428
O -1.5338602843 4.79241869652 -6.4435137
Mg 0.57108920076 -7.85233918872 -2.2637058
Mg -7.85584176156 0.57452189652 -2.2636401
Mg -3.6338961344 -3.63069455112 -6.422742
O 6.89041181088 -1.53107644716 -4.3472586
O -1.5373677852 -7.84246099572 -2.209824
O -1.5344943588 6.89368554572 -4.3473198
O -7.84588143456 -1.53394734636 -2.2099044
Mg -5.74258082112 -5.73910516298 -2.2685451
O 4.7845512702 2.68374095964 -6.440751
O 2.6805700818 4.78771052292 -6.4407504
O 0.57342003732 0.5763829266 -8.5269864
O 6.88720807944 2.68621698384 -4.350183
O 2.68286389488 6.89058644042 -4.3502676
O 2.684312865 -7.8452425584 -2.2074954
O -7.84869799896 2.68776527292 -2.2075059
Mg 4.78455556644 -5.74088304818 -4.3343031
Mg -5.74427151792 4.7879137098 -4.3342743
O 0.57433538916 -5.74562420168 -6.4435407
O -5.74875274896 0.5775245892 -6.4434906
O 6.88702157208 4.7925656532 -2.2092615
O 4.78907975988 6.89047625452 -2.2093218
Mg 0.57342003732 -1.52961698916 -8.5269864
Mg -1.5325800048 0.5763829266 -8.5269864
Mg -1.5315331122 -5.73655888258 -6.4240857
Mg -5.73974214372 -1.52832495816 -6.4240683
O -1.5325800048 -1.52961698916 -8.5269864
Mg 4.78521769284 -3.63351793896 -6.4272132
Mg -3.6367199014 4.78841068368 -6.4271922
Mg 6.89616043272 -3.63093615144 -4.330725
Mg -3.6343506514 6.89953070662 -4.3307376
Mg -3.6377276224 -7.84819167444 -2.2687788
Mg -7.85164888404 -3.634290504 -2.2688622
Mg 2.67941995308 0.5763829266 -8.5269864
Mg 0.57342003732 2.68238296872 -8.5269864
O 0.57474694368 -7.8467879412 -4.3473729
O -7.85005459992 0.57810533976 -4.3473135

Mg 2.67718767732 -5.74156362308 -6.4272765
Mg -5.74476179472 2.68037498196 -6.427254
O 2.67941995308 -1.52961698916 -8.5269864
O -1.5325800048 2.68238296872 -8.5269864
Mg -1.5307016634 -7.84899254412 -4.3326096
Mg -7.8523206138 -1.52732519784 -4.3326453
O -5.74519950576 -5.74184629048 -4.3492248
Mg 6.89341917888 -5.73910516298 -2.2685451
Mg -5.74258082112 6.89689483702 -2.2685451
O 0.57342003732 -3.63561703128 -8.5269864
O -3.6385800469 0.5763829266 -8.5269864
O 2.67941995308 2.68238296872 -8.5269864
Mg 4.78372247496 4.78689726996 -6.4276134
Mg 2.67456810816 -7.85252936052 -4.33083
Mg -7.85589508548 2.67791096196 -4.3307847
O -3.6400382413 -5.74074316768 -6.4407837
O -5.74394828904 -3.63684247058 -6.4407321
Mg -1.5325800048 -3.63561703128 -8.5269864
Mg -3.6385800469 -1.52961698916 -8.5269864
Mg 6.89172848208 4.7879137098 -4.3342743
Mg 4.78455556644 6.89511695182 -4.3343031
O 6.88724725104 0.5775245892 -6.4434906
O 0.57433538916 6.89037579832 -6.4435407
Mg 4.78269137736 -7.84982866824 -2.2686813
Mg -7.85330862264 4.78617133176 -2.2686813
Mg 6.89625785628 -1.52832495816 -6.4240683
Mg -1.5315331122 6.89944111742 -6.4240857
Mg 2.67941995308 -3.63561703128 -8.5269864
Mg -3.6385800469 2.68238296872 -8.5269864
O -3.6426078983 -7.84350220212 -4.3501473
O -7.84685592288 -3.63921033058 -4.3502067
O 4.7854199952 0.5763829266 -8.5269864
O 0.57342003732 4.78838301084 -8.5269864
Mg 6.89123820528 2.68037498196 -6.427254
Mg 2.67718767732 6.89443637692 -6.4272765
O 4.78635013116 -5.74257008048 -6.4416354
O -5.7457427274 4.7895285906 -6.4416222
Mg 4.7854199952 -1.52961698916 -8.5269864
Mg -1.5325800048 4.78838301084 -8.5269864
O -3.6385800469 -3.63561703128 -8.5269864
O 6.89080049424 -5.74184629048 -4.3492248
O -5.74897842792 -7.8434343468 -2.2092615
O -5.74519950576 6.89415370952 -4.3492248
O -7.84692024012 -5.74552374548 -2.2093218
Mg 6.89341917888 6.89689483702 -2.2685451
O 6.89205171096 -3.63684247058 -6.4407321
O -3.6400382413 6.89525683232 -6.4407837
Mg 4.7854199952 2.68238296872 -8.5269864
Mg 2.67941995308 4.78838301084 -8.5269864
Mg 0.57201352416 -7.85261225268 -6.4241013
Mg -7.85577099996 0.57519400536 -6.4240563
O 4.78546510572 -7.8472005066 -4.3492701
O -7.85053489428 4.7887994934 -4.3492701
O -1.5338602843 -7.84358130348 -6.4435137
O -7.84673158464 -1.53066969432 -6.4435428
Mg 0.57342003732 -5.74161707338 -8.5269864
Mg -5.74457996268 0.5763829266 -8.5269864

Mg -5.74310711052 -5.73990148368 -6.4275927
O -1.5325800048 -5.74161707338 -8.5269864
O -5.74457996268 -1.52961698916 -8.5269864
O 4.7854199952 -3.63561703128 -8.5269864
O -3.6385800469 4.78838301084 -8.5269864
O 2.6805700818 -7.84828947708 -6.4407504
O -7.8514487298 2.68374095964 -6.440751
O 6.8902572726 4.7895285906 -6.4416222
O 4.78635013116 6.89342991952 -6.4416354
O 2.67941995308 -5.74161707338 -8.5269864
O -5.74457996268 2.68238296872 -8.5269864
Mg -5.74427151792 -7.8480862902 -4.3342743
Mg -7.85144443356 -5.74088304818 -4.3343031
Mg 0.57342003732 0.5763829266 -10.6329864
O 6.88702157208 -7.8434343468 -2.2092615
O 6.89080049424 6.89415370952 -4.3492248
O -7.84692024012 6.89047625452 -2.2093218
O 0.57342003732 -1.52961698916 -10.6329864
O -1.5325800048 0.5763829266 -10.6329864
Mg -3.6367199014 -7.84758931632 -6.4271922
Mg -7.85078230716 -3.63351793896 -6.4272132
Mg -1.5325800048 -1.52961698916 -10.6329864
O 4.7854199952 4.78838301084 -8.5269864
Mg -3.6385800469 -5.74161707338 -8.5269864
Mg -5.74457996268 -3.63561703128 -8.5269864
Mg 6.89142003732 0.5763829266 -8.5269864
Mg 0.57342003732 6.89438292662 -8.5269864
O 2.67941995308 0.5763829266 -10.6329864
O 0.57342003732 2.68238296872 -10.6329864
Mg 6.89289288948 -5.73990148368 -6.4275927
Mg -5.74310711052 6.89609851632 -6.4275927
O 6.89142003732 -1.52961698916 -8.5269864
Mg 2.67941995308 -1.52961698916 -10.6329864
O -1.5325800048 6.89438292662 -8.5269864
Mg -1.5325800048 2.68238296872 -10.6329864
Mg 4.78372247496 -7.84910273004 -6.4276134
Mg -7.85227752504 4.78689726996 -6.4276134
Mg 0.57342003732 -3.63561703128 -10.6329864
Mg -3.6385800469 0.5763829266 -10.6329864
O 6.89142003732 2.68238296872 -8.5269864
O 2.67941995308 6.89438292662 -8.5269864
Mg 2.67941995308 2.68238296872 -10.6329864
Mg 6.89172848208 -7.8480862902 -4.3342743
Mg -7.85144443356 6.89511695182 -4.3343031
Mg -7.85330862264 -7.84982866824 -2.2686813
Mg 4.7854199952 -5.74161707338 -8.5269864
O -1.5325800048 -3.63561703128 -10.6329864
Mg -5.74457996268 4.78838301084 -8.5269864
O -3.6385800469 -1.52961698916 -10.6329864
Mg 6.89142003732 -3.63561703128 -8.5269864
O 2.67941995308 -3.63561703128 -10.6329864
Mg -3.6385800469 6.89438292662 -8.5269864
O -3.6385800469 2.68238296872 -10.6329864
O 0.57342003732 -7.84761698916 -8.5269864
O -7.8505800048 0.5763829266 -8.5269864
O -5.7457427274 -7.8464714094 -6.4416222
O -7.84964986884 -5.74257008048 -6.4416354

Mg 4.7854199952 0.5763829266 -10.6329864
Mg 0.57342003732 4.78838301084 -10.6329864
Mg 6.89289288948 6.89609851632 -6.4275927
Mg -1.5325800048 -7.84761698916 -8.5269864
Mg -7.8505800048 -1.52961698916 -8.5269864
O 4.7854199952 -1.52961698916 -10.6329864
O -1.5325800048 4.78838301084 -10.6329864
O -5.74457996268 -5.74161707338 -8.5269864
Mg -3.6385800469 -3.63561703128 -10.6329864
Mg 2.67941995308 -7.84761698916 -8.5269864
Mg -7.8505800048 2.68238296872 -8.5269864
O -7.85053489428 -7.8472005066 -4.3492701
Mg 6.89142003732 4.78838301084 -8.5269864
Mg 4.7854199952 6.89438292662 -8.5269864
O 4.7854199952 2.68238296872 -10.6329864
O 2.67941995308 4.78838301084 -10.6329864
O 0.57342003732 -5.74161707338 -10.6329864
O -5.74457996268 0.5763829266 -10.6329864
O -3.6385800469 -7.84761698916 -8.5269864
O -7.8505800048 -3.63561703128 -8.5269864
Mg -1.5325800048 -5.74161707338 -10.6329864
Mg -5.74457996268 -1.52961698916 -10.6329864
Mg 4.7854199952 -3.63561703128 -10.6329864
Mg -3.6385800469 4.78838301084 -10.6329864
O 6.8902572726 -7.8464714094 -6.4416222
O -7.84964986884 6.89342991952 -6.4416354
O 6.89142003732 -5.74161707338 -8.5269864
Mg 2.67941995308 -5.74161707338 -10.6329864
O -5.74457996268 6.89438292662 -8.5269864
Mg -5.74457996268 2.68238296872 -10.6329864
O 4.7854199952 -7.84761698916 -8.5269864
O -7.8505800048 4.78838301084 -8.5269864
Mg 4.7854199952 4.78838301084 -10.6329864
O -3.6385800469 -5.74161707338 -10.6329864
O -5.74457996268 -3.63561703128 -10.6329864
O 6.89142003732 0.5763829266 -10.6329864
O 0.57342003732 6.89438292662 -10.6329864
Mg 6.89142003732 -1.52961698916 -10.6329864
Mg -1.5325800048 6.89438292662 -10.6329864
Mg -7.85227752504 -7.84910273004 -6.4276134
Mg -5.74457996268 -7.84761698916 -8.5269864
Mg -7.8505800048 -5.74161707338 -8.5269864
O 6.89142003732 6.89438292662 -8.5269864
Mg 6.89142003732 2.68238296872 -10.6329864
Mg 2.67941995308 6.89438292662 -10.6329864
O 4.7854199952 -5.74161707338 -10.6329864
O -5.74457996268 4.78838301084 -10.6329864
O 6.89142003732 -3.63561703128 -10.6329864
O -3.6385800469 6.89438292662 -10.6329864
Mg 0.57342003732 -7.84761698916 -10.6329864
Mg -7.8505800048 0.5763829266 -10.6329864
O -1.5325800048 -7.84761698916 -10.6329864
O -7.8505800048 -1.52961698916 -10.6329864
Mg -5.74457996268 -5.74161707338 -10.6329864
Mg 6.89142003732 -7.84761698916 -8.5269864
O 2.67941995308 -7.84761698916 -10.6329864
Mg -7.8505800048 6.89438292662 -8.5269864

O -7.8505800048 2.68238296872 -10.6329864
O 6.89142003732 4.78838301084 -10.6329864
O 4.7854199952 6.89438292662 -10.6329864
Mg -3.6385800469 -7.84761698916 -10.6329864
Mg -7.8505800048 -3.63561703128 -10.6329864
Mg 6.89142003732 -5.74161707338 -10.6329864
Mg -5.74457996268 6.89438292662 -10.6329864
O -7.8505800048 -7.84761698916 -8.5269864
Mg 4.7854199952 -7.84761698916 -10.6329864
Mg -7.8505800048 4.78838301084 -10.6329864
O -5.74457996268 -7.84761698916 -10.6329864
O -7.8505800048 -5.74161707338 -10.6329864
Mg 6.89142003732 6.89438292662 -10.6329864
O 6.89142003732 -7.84761698916 -10.6329864
O -7.8505800048 6.89438292662 -10.6329864
Mg -7.8505800048 -7.84761698916 -10.6329864

Co/MgO(001).O_2^-, structure II
Mg 0.0 0.0 0.0
Mg 0.0 0.0 4.212
Mg 0.00332592156 0.01566459648 8.3969667
Mg 0.0 2.10600004212 2.106
Mg 12.6345828726 2.10458278836 6.2903229
Mg 0.0 4.21199995788 0.0
Mg 0.0 4.21199995788 4.212
Mg 0.00777202452 4.20284593404 8.3805039
Mg 0.0 6.318 2.106
Mg 0.00105839136 6.31956964392 6.3029964
Mg 0.00148624632 6.32602739808 10.4682825
Mg 0.0 8.42400004212 0.0
Mg 0.0 8.42400004212 4.212
Mg 0.00139071816 8.42392940688 8.4006855
Mg 0.0 10.5299999579 2.106
Mg 0.00060766524 10.5315616411 6.3040263
Mg 0.00324214488 10.5199489044 10.473456
Mg 2.10600004212 0.0 2.106
Mg 2.10761517564 12.6329364018 6.3012996
Mg 2.09749740408 0.00965718936 10.4632173
Mg 2.10600004212 2.10600004212 0.0
Mg 2.10600004212 2.10600004212 4.212
Mg 2.09684614464 2.11377194028 8.3805039
Mg 2.10600004212 4.21199995788 2.106
Mg 2.10967977168 4.2156798138 6.3026931
Mg 2.07682920432 4.18282899372 10.5695424
Mg 2.10600004212 6.318 0.0
Mg 2.10600004212 6.318 4.212
Mg 2.10384762588 6.3019939788 8.4123726
Mg 2.10600004212 8.42400004212 2.106
Mg 2.10711895992 8.421622326 6.3062853
Mg 2.10357254016 8.42087124216 10.4788887
Mg 2.10600004212 10.5299999579 0.0
Mg 2.10600004212 10.5299999579 4.212
Mg 2.10549397032 10.5323607418 8.400402
Mg 4.21199995788 0.0 0.0
Mg 4.21199995788 0.0 4.212
Mg 4.20788125368 0.0022852206 8.3997408
Mg 4.21199995788 2.10600004212 2.106

Mg 4.21356972816 2.10705843348 6.3029964
Mg 4.22002748232 2.10748616208 10.4682825
Mg 4.21199995788 4.21199995788 0.0
Mg 4.21199995788 4.21199995788 4.212
Mg 4.1959941894 4.20984754164 8.4123726
Mg 4.21199995788 6.318 2.106
Mg 4.21134288588 6.31734280164 6.3076668
Mg 4.20067153116 6.30667144692 10.484985
Mg 4.21199995788 8.42400004212 0.0
Mg 4.21199995788 8.42400004212 4.212
Mg 4.21014221316 8.42176460736 8.4088395
Mg 4.21199995788 10.5299999579 2.106
Mg 4.21289408124 10.5287810893 6.3054951
Mg 4.2068257686 10.535515951 10.4794044
Mg 6.318 0.0 2.106
Mg 6.31921798404 12.6355597618 6.3049716
Mg 6.31844630352 0.0046336212 10.4767209
Mg 6.318 2.10600004212 0.0
Mg 6.318 2.10600004212 4.212
Mg 6.31792949112 2.10739063392 8.4006858
Mg 6.318 4.21199995788 2.106
Mg 6.31562241024 4.21311900204 6.3062853
Mg 6.3148713264 4.20957245592 10.4788887
Mg 6.318 6.318 0.0
Mg 6.318 6.318 4.212
Mg 6.3157646916 6.31614200256 8.4088395
Mg 6.318 8.42400004212 2.106
Mg 6.3178193052 8.4238190946 6.3072636
Mg 6.31756519524 8.423565111 10.4816424
Mg 6.318 10.5299999579 0.0
Mg 6.318 10.5299999579 4.212
Mg 6.31901997792 10.5314666184 8.4079677
Mg 8.42400004212 0.0 0.0
Mg 8.42400004212 0.0 4.212
Mg 8.43082272396 0.00342283968 8.4039834
Mg 8.42400004212 2.10600004212 2.106
Mg 8.425561599 2.10660770736 6.3040263
Mg 8.41394886228 2.10924206064 10.473456
Mg 8.42400004212 4.21199995788 0.0
Mg 8.42400004212 4.21199995788 4.212
Mg 8.426360826 4.21149401244 8.4004023
Mg 8.42400004212 6.318 2.106
Mg 8.42278117356 6.318893997 6.3054951
Mg 8.4295160352 6.31282581072 10.4794044
Mg 8.42400004212 8.42400004212 0.0
Mg 8.42400004212 8.42400004212 4.212
Mg 8.425466829 8.42501989368 8.4079677
Mg 8.42400004212 10.5299999579 2.106
Mg 8.4243081078 10.5303080236 6.3060858
Mg 8.4334603626 10.539460152 10.4814909
Mg 10.5299999579 0.0 2.106
Mg 10.5296605549 -0.00033952932 6.3034356
Mg 10.5525815008 0.02258141652 10.4759319
Mg 10.5299999579 2.10600004212 0.0
Mg 10.5299999579 2.10600004212 4.212
Mg 10.5456646807 2.10932583732 8.3969664
Mg 10.5299999579 4.21199995788 2.106

Mg 10.526936486 4.2136150914 6.3012996
Mg 10.5396572736 4.20349731984 10.463217
Mg 10.5299999579 6.318 0.0
Mg 10.5299999579 6.318 4.212
Mg 10.5322853048 6.31388116944 8.3997408
Mg 10.5299999579 8.42400004212 2.106
Mg 10.529559846 8.4252178998 6.3049716
Mg 10.5346338318 8.42444621928 10.4767209
Mg 10.5299999579 10.5299999579 0.0
Mg 10.5299999579 10.5299999579 4.212
Mg 10.5334229239 10.5368225134 8.4039831
O 0.0 0.0 2.106
O 12.6356512464 0.00265520268 6.3036471
O 0.00881853804 0.08114005224 10.4959668
O 0.0 2.10600004212 0.0
O 0.0 2.10600004212 4.212
O 12.6289257354 2.0989255248 8.4062274
O 0.0 4.21199995788 2.106
O 12.6357212498 4.20845922432 6.3014979
O 12.6177584177 4.14912890808 10.4189994
O 0.0 6.318 0.0
O 0.0 6.318 4.212
O 12.6344447611 6.31848876048 8.3953218
O 0.0 8.42400004212 2.106
O 12.6356861218 8.42615346924 6.3088722
O 12.6353760343 8.42505754896 10.5287346
O 0.0 10.5299999579 0.0
O 0.0 10.5299999579 4.212
O 0.00027091584 10.5378876018 8.4028881
O 2.10600004212 0.0 0.0
O 2.10600004212 0.0 4.212
O 2.1085320438 12.6275129042 8.3798373
O 2.10600004212 2.10600004212 2.106
O 2.10245930856 2.10572129196 6.3014979
O 2.04312911868 2.0877584598 10.4189994
O 2.10600004212 4.21199995788 0.0
O 2.10600004212 4.21199995788 4.212
O 2.10846393576 4.21446385152 8.3904711
O 2.10600004212 6.318 2.106
O 2.10577752216 6.319784835 6.3070539
O 2.08654009668 6.30555088644 10.5408861
O 2.10600004212 8.42400004212 0.0
O 2.10600004212 8.42400004212 4.212
O 2.10510402336 8.42291776872 8.4017373
O 2.10600004212 10.5299999579 2.106
O 2.10609607572 10.5265239206 6.3057561
O 2.0911733388 10.5422476536 10.5328857
O 4.21199995788 0.0 2.106
O 4.21477621344 12.6357920114 6.3055584
O 4.19997377124 0.01625773032 10.531707
O 4.21199995788 2.10600004212 0.0
O 4.21199995788 2.10600004212 4.212
O 4.21248884472 2.10444467688 8.3953218
O 4.21199995788 4.21199995788 2.106
O 4.21378491924 4.21177756428 6.3070539
O 4.19955097068 4.19254001244 10.5408861
O 4.21199995788 6.318 0.0

O 4.21199995788 6.318 4.212
O 4.21077401316 6.31677380256 8.4062613
O 4.21199995788 8.42400004212 2.106
O 4.21300553076 8.42367100068 6.3109566
O 4.205949462 8.42430406428 10.5376011
O 4.21199995788 10.5299999579 0.0
O 4.21199995788 10.5299999579 4.212
O 4.21235730396 10.5307509154 8.4029115
O 6.318 0.0 0.0
O 6.318 0.0 4.212
O 6.32051848116 0.0011239722 8.400681
O 6.318 2.10600004212 2.106
O 6.32015342712 2.10568603752 6.3088719
O 6.31905750684 2.10537582372 10.5287346
O 6.318 4.21199995788 0.0
O 6.318 4.21199995788 4.212
O 6.31691797932 4.21110381276 8.4017373
O 6.318 6.318 2.106
O 6.31767108492 6.31900532016 6.3109566
O 6.31830414852 6.31194937776 10.5376011
O 6.318 8.42400004212 0.0
O 6.318 8.42400004212 4.212
O 6.31875070476 8.42475062052 8.4043263
O 6.318 10.5299999579 2.106
O 6.31900620468 10.529749386 6.3107766
O 6.3201601242 10.5387147544 10.5375675
O 8.42400004212 0.0 2.106
O 8.42402683044 0.00057860244 6.3080151
O 8.44216757748 0.02072708352 10.5369828
O 8.42400004212 2.10600004212 0.0
O 8.42400004212 2.10600004212 4.212
O 8.43188768604 2.1062708316 8.4028881
O 8.42400004212 4.21199995788 2.106
O 8.42052400488 4.21209599148 6.3057561
O 8.43624773784 4.19717325456 10.532886
O 8.42400004212 6.318 0.0
O 8.42400004212 6.318 4.212
O 8.4247509996 6.31835709336 8.4029115
O 8.42400004212 8.42400004212 2.106
O 8.42374947024 8.42500612044 6.3107769
O 8.4327148386 8.42616003996 10.5375675
O 8.42400004212 10.5299999579 0.0
O 8.42400004212 10.5299999579 4.212
O 8.42728818204 10.5332880978 8.404917
O 10.5299999579 0.0 0.0
O 10.5299999579 0.0 4.212
O 10.5304690062 0.0004687956 8.3923134
O 10.5299999579 2.10600004212 2.106
O 10.5326551606 2.10565116216 6.3036471
O 10.6111405156 2.1148184538 10.4959659
O 10.5299999579 4.21199995788 0.0
O 10.5299999579 4.21199995788 4.212
O 10.5215128621 4.21453208592 8.3798373
O 10.5299999579 6.318 2.106
O 10.5297920957 6.3207761292 6.3055584
O 10.5462578146 6.30597381336 10.5317073
O 10.5299999579 8.42400004212 0.0

O 10.5299999579 8.42400004212 4.212
O 10.5311240564 8.42651827056 8.400681
O 10.5299999579 10.5299999579 2.106
O 10.5305786867 10.5300267462 6.3080148
O 10.5507270414 10.5481673669 10.5369825
O 0.16980269436 2.27580248376 12.3455031
O 1.05504736896 3.16104728472 12.7744983
Co 0.03839840316 2.14439831892 10.5405684

Co/MgO(001).O_2^-, structure III
Mg 0.0 0.0 0.0
Mg 0.0 0.0 4.212
Mg 0.000382239 -0.00478992852 8.3955762
Mg 0.0 2.10600004212 2.106
Mg 0.00062914644 2.10635372376 6.2887848
Mg 0.0 4.21199995788 0.0
Mg 0.0 4.21199995788 4.212
Mg 0.0022353084 4.21779659652 8.3966187
Mg 0.0 6.318 2.106
Mg 0.00201885372 6.31964861892 6.3027513
Mg 0.00254703852 6.33799950264 10.4711559
Mg 0.0 8.42400004212 0.0
Mg 0.0 8.42400004212 4.212
Mg 0.00057316896 8.42315936904 8.396445
Mg 0.0 10.5299999579 2.106
Mg -7.316244e-05 10.5280226766 6.3015402
Mg 0.00064051884 10.5103968466 10.4693814
Mg 2.10600004212 0.0 2.106
Mg 2.10753203076 -0.00174199896 6.3018972
Mg 2.10766673052 12.6159806588 10.4704623
Mg 2.10600004212 2.10600004212 0.0
Mg 2.10600004212 2.10600004212 4.212
Mg 2.10613284648 2.1075325362 8.3907576
Mg 2.10600004212 4.21199995788 2.106
Mg 2.10848453244 4.21522314876 6.3038766
Mg 2.11312030176 4.23656573184 10.475007
Mg 2.10600004212 6.318 0.0
Mg 2.10600004212 6.318 4.212
Mg 2.11686535944 6.32245014648 8.4076992
Mg 2.10600004212 8.42400004212 2.106
Mg 2.1079432062 8.42304109608 6.3041238
Mg 2.11450293288 8.42357547252 10.469622
Mg 2.10600004212 10.5299999579 0.0
Mg 2.10600004212 10.5299999579 4.212
Mg 2.10845003616 10.5255864558 8.3979408
Mg 4.21199995788 0.0 0.0
Mg 4.21199995788 0.0 4.212
Mg 4.2119856792 -0.00094353012 8.4008031
Mg 4.21199995788 2.10600004212 2.106
Mg 4.21298417592 2.1076778502 6.3037335
Mg 4.22775464904 2.1075350634 10.474029
Mg 4.21199995788 4.21199995788 0.0
Mg 4.21199995788 4.21199995788 4.212
Mg 4.2134590368 4.22196635016 8.4102135
Mg 4.21199995788 6.318 2.106
Mg 4.21325660808 6.31826333424 6.3080817
Mg 4.22795278152 6.33346039872 10.5530205

Mg 4.21199995788 8.42400004212 0.0
Mg 4.21199995788 8.42400004212 4.212
Mg 4.21942600872 8.41804532712 8.4097698
Mg 4.21199995788 10.5299999579 2.106
Mg 4.21394779728 10.5289174318 6.305424
Mg 4.21392808512 10.5221475684 10.4723001
Mg 6.318 0.0 2.106
Mg 6.31772023896 -0.00264623112 6.3051882
Mg 6.31726357392 12.6332520491 10.4769879
Mg 6.318 2.10600004212 0.0
Mg 6.318 2.10600004212 4.212
Mg 6.31767222216 2.10577575312 8.4000489
Mg 6.318 4.21199995788 2.106
Mg 6.317383995 4.21292554488 6.3055572
Mg 6.31831438368 4.2163672122 10.4720595
Mg 6.318 6.318 0.0
Mg 6.318 6.318 4.212
Mg 6.31292020164 6.32295305928 8.4099201
Mg 6.318 8.42400004212 2.106
Mg 6.31811524032 8.42381871552 6.3086943
Mg 6.3054941508 8.40998229552 10.5571368
Mg 6.318 10.5299999579 0.0
Mg 6.318 10.5299999579 4.212
Mg 6.3162947718 10.5198797855 8.4124137
Mg 8.42400004212 0.0 0.0
Mg 8.42400004212 0.0 4.212
Mg 8.42366645172 -0.00252922176 8.4021123
Mg 8.42400004212 2.10600004212 2.106
Mg 8.42321926368 2.10558077964 6.3023763
Mg 8.41009690404 2.105779923 10.4722098
Mg 8.42400004212 4.21199995788 0.0
Mg 8.42400004212 4.21199995788 4.212
Mg 8.42376892968 4.21353573732 8.400327
Mg 8.42400004212 6.318 2.106
Mg 8.42270548392 6.31902945492 6.305352
Mg 8.41779374436 6.3197349228 10.4719617
Mg 8.42400004212 8.42400004212 0.0
Mg 8.42400004212 8.42400004212 4.212
Mg 8.41292382996 8.42178482496 8.4117267
Mg 8.42400004212 10.5299999579 2.106
Mg 8.42172000228 10.5280672817 6.3055944
Mg 8.41786425324 10.5225886912 10.4793288
Mg 10.5299999579 0.0 2.106
Mg 10.5288569053 -0.00244430784 6.3019542
Mg 10.5285789133 -0.0201708468 10.4707851
Mg 10.5299999579 2.10600004212 0.0
Mg 10.5299999579 2.10600004212 4.212
Mg 10.5318307879 2.10586281516 8.3897397
Mg 10.5299999579 4.21199995788 2.106
Mg 10.5296263114 4.21431146136 6.3019143
Mg 10.5287656734 4.23120680424 10.4703519
Mg 10.5299999579 6.318 0.0
Mg 10.5299999579 6.318 4.212
Mg 10.5294720258 6.32114611128 8.3982234
Mg 10.5299999579 8.42400004212 2.106
Mg 10.5271694939 8.4234937176 6.3037062
Mg 10.5250534693 8.4226944906 10.4739873

Mg 10.5299999579 10.5299999579 0.0
Mg 10.5299999579 10.5299999579 4.212
Mg 10.5277973767 10.525612486 8.3996463
O 0.0 0.0 2.106
O -0.00033535944 0.00298386504 6.3059193
O -0.00124894224 0.0031444686 10.5205857
O 0.0 2.10600004212 0.0
O 0.0 2.10600004212 4.212
O 9.931896e-05 2.10546516024 8.3899452
O 0.0 4.21199995788 2.106
O 0.00049697388 4.20864636348 6.3062316
O -0.00020609316 4.2070728024 10.5216408
O 0.0 6.318 0.0
O 0.0 6.318 4.212
O -0.00015517008 6.31638600372 8.3948901
O 0.0 8.42400004212 2.106
O 12.6340958812 8.42436218988 6.3038103
O -0.00160173936 8.42549298552 10.5255585
O 0.0 10.5299999579 0.0
O 0.0 10.5299999579 4.212
O 12.6344338942 10.5320937431 8.3938698
O 2.10600004212 0.0 0.0
O 2.10600004212 0.0 4.212
O 2.10181335624 0.00527312916 8.3971941
O 2.10600004212 2.10600004212 2.106
O 2.10248799228 2.10610264644 6.3059772
O 2.04291809748 2.10491991684 10.5327009
O 2.10600004212 4.21199995788 0.0
O 2.10600004212 4.21199995788 4.212
O 2.10202185024 4.20639336468 8.3996655
O 2.10600004212 6.318 2.106
O 2.1053382948 6.31602916308 6.3066471
O 2.09508569712 6.3163566882 10.5303162
O 2.10600004212 8.42400004212 0.0
O 2.10600004212 8.42400004212 4.212
O 2.0973081168 8.42577881184 8.3878062
O 2.10600004212 10.5299999579 2.106
O 2.10470409396 10.5324619561 6.30495
O 2.09566341504 10.536618821 10.5273144
O 4.21199995788 0.0 2.106
O 4.21103633652 0.00379231632 6.3063285
O 4.19761816812 0.01517924772 10.5339036
O 4.21199995788 2.10600004212 0.0
O 4.21199995788 2.10600004212 4.212
O 4.20254014284 2.10510983592 8.4031626
O 4.21199995788 4.21199995788 2.106
O 4.210804845 4.20915496248 6.3079311
O 4.19927866488 4.19118063156 10.5355536
O 4.21199995788 6.318 0.0
O 4.21199995788 6.318 4.212
O 4.20779975148 6.31100306772 8.4028602
O 4.21199995788 8.42400004212 2.106
O 4.21222487868 8.42412058956 6.3075996
O 4.19126908356 8.44399145772 10.473786
O 4.21199995788 10.5299999579 0.0
O 4.21199995788 10.5299999579 4.212
O 4.20939125568 10.5421166183 8.3913042

O 6.318 0.0 0.0
O 6.318 0.0 4.212
O 6.3191220768 0.00358887672 8.3993844
O 6.318 2.10600004212 2.106
O 6.318616005 2.10366124488 6.308085
O 6.3202403628 2.10363609924 10.5290835
O 6.318 4.21199995788 0.0
O 6.318 4.21199995788 4.212
O 6.32032704576 4.19869841976 8.3917245
O 6.318 6.318 2.106
O 6.31843354116 6.31634430492 6.307875
O 6.33949674228 6.28841558592 10.4732154
O 6.318 8.42400004212 0.0
O 6.318 8.42400004212 4.212
O 6.321951909 8.42742439812 8.4021858
O 6.318 10.5299999579 2.106
O 6.31890056772 10.5318871445 6.3079098
O 6.31865884104 10.5442915266 10.5322611
O 8.42400004212 0.0 2.106
O 8.42544875952 0.00153767484 6.3078744
O 8.43902626788 0.01404516672 10.5357672
O 8.42400004212 2.10600004212 0.0
O 8.42400004212 2.10600004212 4.212
O 8.4349646784 2.10368272608 8.4019851
O 8.42400004212 4.21199995788 2.106
O 8.42626656144 4.20847198668 6.3062556
O 8.44328750616 4.1919064434 10.5323133
O 8.42400004212 6.318 0.0
O 8.42400004212 6.318 4.212
O 8.43471018936 6.31263968244 8.390949
O 8.42400004212 8.42400004212 2.106
O 8.42471271252 8.42467556268 6.3075198
O 8.43157102788 8.42401217268 10.5303849
O 8.42400004212 10.5299999579 0.0
O 8.42400004212 10.5299999579 4.212
O 8.42689166436 10.5347215256 8.3998281
O 10.5299999579 0.0 0.0
O 10.5299999579 0.0 4.212
O 10.5331686876 0.00363701988 8.3977539
O 10.5299999579 2.10600004212 2.106
O 10.5337246716 2.10537721368 6.3056319
O 10.5925146725 2.10414217104 10.5314022
O 10.5299999579 4.21199995788 0.0
O 10.5299999579 4.21199995788 4.212
O 10.5349015886 4.20628406328 8.3971194
O 10.5299999579 6.318 2.106
O 10.5323924581 6.31631220948 6.3049086
O 10.5370890066 6.31457349588 10.5285126
O 10.5299999579 8.42400004212 0.0
O 10.5299999579 8.42400004212 4.212
O 10.530577044 8.42470323552 8.3949408
O 10.5299999579 10.5299999579 2.106
O 10.5299796139 10.5317228765 6.3065832
O 10.5357890149 10.5327299657 10.5303402
O 4.84044446808 6.92647558668 12.9255276
O 5.74304456232 7.83265470156 12.9090567
Co 0.0017924166 2.10750258888 10.4960172
